# A scoring system is an effective tool for predicting central lymph node metastasis in papillary thyroid microcarcinoma: a case-control study

**DOI:** 10.1186/s12957-016-0808-6

**Published:** 2016-02-24

**Authors:** Ye-feng Cai, Qing-xuan Wang, Chun-jue Ni, Xiang-jian Zhang, En-dong Chen, Si-yang Dong, Hua-min Zheng, Xiao-hua Zhang, Quan Li

**Affiliations:** Department of Oncology, the First Affiliated Hospital of Wenzhou Medical University, Wenzhou Medical University, No. 2 Fuxue Lane, Wenzhou, 325000 China; Department of Anesthesiology, the First Affiliated Hospital of Wenzhou Medical University, Wenzhou, 325000 China; Department of Diagnostic Ultrasound, the First Affiliated Hospital of Wenzhou Medical University, Wenzhou, 325000 China

**Keywords:** Thyroid, Papillary thyroid microcarcinoma, Central lymph node, Central lymph node dissection, Predictive factor, Scoring system

## Abstract

**Background:**

The purpose of this study was to evaluate the clinicopathologic and ultrasonographic (US) characteristics and establish an effective scoring system for predicting central lymph node metastasis (CLNM) in papillary thyroid microcarcinoma (PTMC).

**Methods:**

A total of 498 patients with PTMC who underwent total thyroidectomy or lobectomy with therapeutic central lymph node dissection (CLND) were enrolled. Univariate and multivariate analyses were performed to find the independent predictors for CLNM based on clinicopathological and US characteristics. Using the standardized regression coefficient, a 10-point score system was constructed in line with these independent predictors. Then, the scoring system was evaluated for the diagnostic value in predicting CLNM.

**Results:**

Tumor location (the lower polo), tumor size (>5 mm), extrathyroidal extension, margin (no well-defined), display of enlarged lymph node, and contact of >25 % with the adjacent capsule were independent predictors for CLNM. Verifying the scoring system, a cutoff value of 5 points was found to be the best prediction for CLNM, the sensitivity and specificity were 64.7 and 80.5 %, respectively, and the positive and negative predictive values were 77.3 and 69.0 %, respectively.

**Conclusions:**

The points ≤ 5 could be considered as a low risk for CLNM, and the points > 5 could be identified as a high risk for CLNM. More advanced diagnostic approaches and prophylactic CLND are needed for patients with the points > 5.

## Background

Papillary thyroid microcarcinoma (PTMC) is defined as a papillary thyroid carcinoma measuring 1.0 cm or less in maximal diameter [1]. In the past, most patients with PTMC were diagnosed from specimens of thyroid removed for benign diseases, such as Graves’ disease, follicular adenoma, and multinodular goiter. In recent years, due to the use of ultrasonography (US) and US-guided fine-needle aspiration cytology (FNAC), the number of patients with PTMC has been increasing. Although PTMC is usually associated with a favorable prognosis, regional lymph node metastasis (LNM), particularly in the central cervical compartment, is not uncommon [2]. The central lymph nodes are considered as the first echelon of lymphatic metastasis and are the level most frequently involved in papillary thyroid carcinoma (PTC) [3]. Recent studies demonstrated that the presence of nodal involvement was commonly associated with local recurrence and cancer-specific mortality [4, 5]. Therefore, it is crucial to detect the metastatic lymph nodes preoperatively for PTMC, especially the central lymph nodes.

Nevertheless, the central lymph node metastasis (CLNM) is frequently diagnosed with microscopic LNM by pathology (metastatic deposits within a lymph node of less than 2 mm in maximal diameter) which is not easily detected preoperatively [6, 7]. In clinical node-negative (cN0) PTMC, the presence of microscopic LNM is frequently detected in surgical specimens, and the prevalence of subclinical CLNM ranges from 30 % to approximately 65 % [2, 8]. Therefore, it is generally agreed that prophylactic central lymph node dissection (pCLND) should be performed to remove microscopic LNM because this treatment reduced local recurrence and cancer-specific mortality. However, pCLND remains a controversial subject. Zetoune et al. [9] showed that pCLND did not greatly reduce local recurrence in thyroid cancer. Carling et al. [10] demonstrated that the pCLND would lead to higher rates of hypoparathyroidism and recurrent laryngeal nerve injury. Moreover, a major argument against pCLND was a possibly increased risk of operative complications and medical cost which added the burden of patients. A prospective randomized controlled single-institution study demonstrated that cN0 patients with PTC treated with pCLND showed a similar outcome. Although one advantage pCLND could reduce necessity to repeat (131) I treatments, the disadvantage was a higher prevalence of permanent hypoparathyroidism. Actually, the different findings on this studies of pCLND could be explained with the variant percents of CLNM in different studies. Almost all clinicians agreed that central lymph node dissection (CLND) should be done with evidence of CLNM preoperatively. Hence, how to evaluate accurately the status of CLNM and choose the CLND as an optimum operation plan for PTMC are very important in thyroid operation.

Our study focused on investigating the relationship between CLNM and the clinicopathological and US characteristics to find the risk predictors for CLNM in PTMC. In addition, using the standardized regression coefficient, we developed a scoring system to identify the risk of CLNM and evaluate the predictive value of the scoring system.

## Methods

### Patient

We conducted a retrospective case-control study involving 498 patients who underwent total thyroidectomy or lobectomy with prophylactic central lymph node at the First Affiliated Hospital of Wenzhou Medical University from January 2009 to December 2012. All patients had no history of thyroid or neck surgery for nonthyroidial cancer, as well as of neck irradiation. The patients were divided into the case group (252 patients) and the control group (246 patients), according to the status of CLNM.

The surgical procedures were performed in the same operation team of the department of surgical oncology. The patients’ clinical data were obtained from their electronic medical records. The research protocol used in this study was approved by the Ethics Committee of the First Affiliated Hospital of Wenzhou Medical University, and informed consent was obtained from each patient.

### Parameters analyzed

Clinicopathologic and US characteristics were used to analyze risk factors of CLNM: a total of 16 selected factors in Table 1. All pathological diagnoses were made by an experienced pathologist. Primary tumor size based on US was measured by the standard of change in echo edge. When multiple PTMC were found in the specimen, the largest tumor or the most suspicious dominant nodule was analyzed. We used the age of 45 years as the cutoff because of its wide use as a clinical factor for prognosis. When multiple PTC were found in the specimen, the largest tumor or the most suspicious dominant nodule was analyzed. US calcification was defined as the hyperechoic spots with or without acoustic shadows or as the simple fine acoustic shadows in ultrasound [11]. ETE was defined as the tumor perimeter in contact with >25 % of the thyroid capsule in a malignant lesion or the loss of the capsule line according to the ultrasound [12]. Tumor location was classified in four areas (upper, middle, lower, and isthmus) based on preoperative ultrasonographic findings. LNM was obtained from the final pathologic reports. All the ultrasound features of the thyroid nodules were retrospectively reviewed by two experienced radiologists.

**Table 1:** Baseline characteristics of papillary thyroid microcarcinoma patients stratified by the status of metastatic central lymph node

Characteristics	Total	The status of metastatic central lymph node		*p* value
		Positive	Negative	
Total	498	252	246	
Sex				0.130
Male (*n*, %)	143 (28.7 %)	80 (31.7 %)	63 (25.6 %)	
Female (*n*, %)	355 (71.3 %)	172 (68.3 %)	183 (74.4 %)	
Age at diagnosis (years)		45.8 ± 9.8	47.2 ± 11.9	0.150
<45 (*n*, %)	251 (50.4 %)	136 (54.0 %)	115 (46.7 %)	0.107
≥45 (*n*, %)	247 (49.6 %)	116 (46.0 %)	131 (53.3 %)	
Tumor size (mm)		6.8 ± 2.1	5.7 ± 2.6	<0.001
≤5 (*n*, %)	165 (33.1 %)	51 (20.2 %)	114 (46.3 %)	<0.001
>5 (*n*, %)	333 (66.9 %)	201 (79.8 %)	132 (53.7 %)	
Tumor location				<0.001
Lower (*n*, %)	141 (28.3 %)	93 (36.9 %)	48 (19.5 %)	
Upper/middle/isthmus (*n*, %)	357 (71.7 %)	159 (63.1 %)	198 (80.5 %)	
No. of nodules				0.416
Single (*n*, %)	431 (86.5 %)	215 (85.3 %)	216 (87.8 %)	
Multiple (*n*, %)	67 (13.5 %)	37 (14.7 %)	30 (12.2 %)	
Hashimoto’s thyroiditis				0.348
Yes (*n*, %)	87 (17.5 %)	48 (19.0 %)	39 (15.9 %)	
No (*n*, %)	411 (82.5 %)	204 (81.0 %)	207 (84.1 %)	
Extrathyroidal extension				<0.001
Yes (*n*, %)	249 (50.0 %)	150 (59.5 %)	99 (40.2 %)	
No (*n*, %)	249 (50.0 %)	102 (40.5 %)	147 (59.8 %)	
Vascularization				0.29
Absence (*n*, %)	435 (87.3 %)	225 (89.3 %)	210 (85.4 %)	
External vascularization (*n*, %)	31 (6.2 %)	15 (6.0 %)	16 (6.5 %)	
Internal vascularization (*n*, %)	32 (6.4 %)	12 (4.8 %)	20 (8.1 %)	
Intact capsule				0.548
Yes (*n*, %)	297 (59.6 %)	147 (58.3 %)	150 (61.0 %)	
No (*n*, %)	201 (40.4 %)	105 (41.7 %)	96 (39.0 %)	
Margin				<0.001
Well-defined (*n*, %)	246 (89.8 %)	99 (39.3 %)	147 (59.8 %)	
No well-defined (*n*, %)	252 (50.6 %)	153 (60.7 %)	99 (40.2 %)	
Shape				0.280
Regular (*n*, %)	372 (74.7 %)	183 (72.6 %)	189 (76.8 %)	
Irregular (*n*, %)	126 (25.3 %)	69 (27.4 %)	57 (23.2 %)	
Composition				0.188
Solid (*n*, %)	435 (87.3 %)	225 (89.3 %)	210 (85.4 %)	
Cystic or mixed (*n*, %)	63 (12.7 %)	27 (10.7 %)	36 (14.6 %)	
Calcification				<0.001
Absence (*n*, %)	237 (47.6 %)	102 (40.5 %)	135 (54.9 %)	
Microcalcification (*n*, %)	207 (41.6 %)	129 (51.2 %)	78 (31.7 %)	
Other calcification (*n*, %)	54 (10.8 %)	21 (8.3 %)	33 (13.4 %)	
Taller than wide				0.916
Yes (*n*, %)	432 (86.7 %)	219 (86.9 %)	213 (86.6 %)	
No (*n*, %)	66 (13.3 %)	33 (13.1 %)	33 (13.4 %)	
Display of enlarged lymph nodes				<0.001
Yes (*n*, %)	267 (53.6 %)	165 (65.5 %)	102 (41.5 %)	
No (*n*, %)	231 (46.4 %)	87 (34.5 %)	144 (58.5 %)	
Contact of >25 % with the adjacent capsule				<0.001
Yes (*n*, %)	153 (30.7 %)	105 (41.7 %)	48 (19.5 %)	
No (*n*, %)	345 (69.3 %)	147 (58.3 %)	198 (80.5 %)	

### Statistical analysis

Data on normal distribution were expressed as mean ± standard variation (SD) and were compared with *t* test. Categorical variables were expressed as percentage and were compared with chi-square test or Fisher’s exact test, as appropriate. Logistic regression analysis was also performed to estimate the odds ratios (OR) of certain parameters. Variables with *p* < 0.05 in the univariate analysis were progressed to a multivariate analysis using forward stepwise selection. The standardized regression coefficient was used to quantify the discriminating power of each factor and to construct a 10-point score system. The best point for the score system was performed by receiver-operating characteristic (ROC) curves. All *p* values were two sided, and a *p* value of <0.05 was considered statistically significant. Statistical analysis was performed with SPSS software version 19.0 (SPSS, Chicago, IL, USA).

## Results

### Univariate analysis

According to the status of metastatic central lymph node, patients were divided into two groups (the case group: CLNM positive; the control group: CLNM negative). Differences in clinicopathological and US features between two groups were compared in Table 1. Seven factors were significantly associated with CLNM: display of enlarged lymph node (*p* < 0.001), contact of >25 % with the adjacent capsule (*p* < 0.001), no well-defined margin (*p* < 0.001), tumor size (*p* < 0.001), tumor location (*p* < 0.001), extrathyroidal extension (*p* < 0.001), and calcification (*p* < 0.001).

### Multivariate analysis

In the multivariate logistic regression, tumor size > 5 mm (OR 2.986; *p* < 0.001; 95 % confidence interval (CI) 1.861–4.790; standardized coefficient regression (Coef) 1.094), the lower polo (OR 3.568; *p* < 0.001; 95 % CI 2.211–5.758; Coef 1.272), extrathyroidal extension (OR 2.631; *p* < 0.001; 95 % CI 1.720–4.023; Coef 0.967), no well-defined margin (OR 2.459; *p* < 0.001; 95 % CI 1.611–3.754; Coef 0.9), display of metastatic lymph node (OR 3.01; *p* < 0.001; 95 % CI 1.954–4.634; Coef 1.102), and contact of >25 % with the adjacent capsule (OR 2.471; *p* < 0.001; 95 % CI 1.556–3.922; Coef 0.904) had highly independent association with central LNM. The relevant information is shown in Table 2.

**Table 2:** Multivariate analysis (logistic regression) and 10-point scoring system

Characteristic	OR	95 % CI	*p* value	Coef	Score
Size	2.986	1.861–4.790	<0.001	1.094	1.75
Tumor location	3.568	2.211–5.758	<0.001	1.272	2.00
Extrathyroidal extension	2.631	1.720–4.023	<0.001	0.967	1.55
Margin	2.459	1.611–3.754	<0.001	0.900	1.45
Display of enlarged lymph nodes	3.010	1.954–4.634	<0.001	1.102	1.75
Contact of >25 % with the adjacent capsule	2.471	1.556–3.922	<0.001	0.904	1.50
					Total 10

*Coef* standardized coefficient regression, *OR* odds ratio, *95 % CI* 95 % confidence interval

### A scoring system

**Fig 1:**
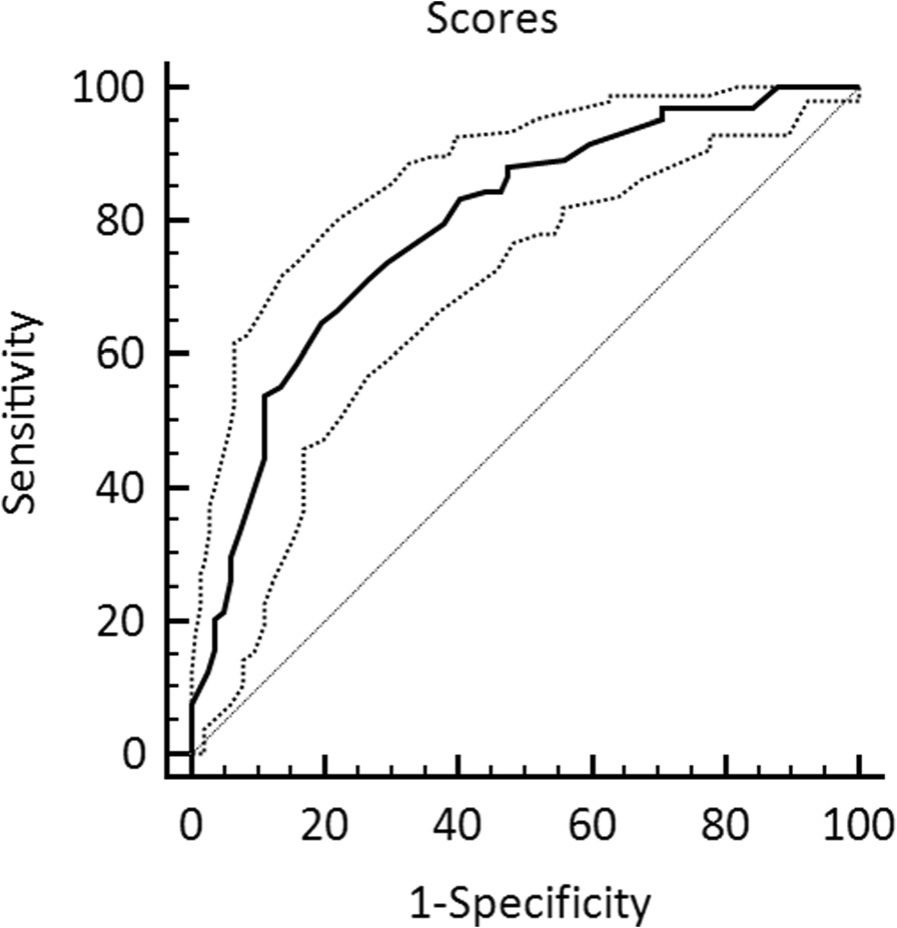
Receiver-operating characteristic (ROC) curve for the scoring system. The area under the ROC curve was 0.791. According to the ROC curve, a cutoff value of 5 points was found to be best for distinguishing between patients without and with central LNM. The sensitivity and specificity were 64.7 and 80.5 %, respectively, and the positive predictive value and negative predictive value were 77.2 and 69.0 %, respectively. The *dotted line* represents the 95 % CI

As shown in Table 2, the standardized regression coefficient is used to quantify the discriminating power (Coef value) of six predictors and construct a 10-point scoring system based on their relative predictive weight. The sum of the points was evaluated to distinguish between patients with and without CLNM. The mean index points were 5.71 ± 1.72 in the CLNM-positive group and 3.55 ± 1.95 in the CLNM-negative group, which showed statistically significant difference (*p* < 0.001). Using ROC curve, the area under the ROC curve was 0.791 (Fig. 1). Then, the cutoff value of 5 was found to be the best points. The sensitivity and specificity for this scoring system were 64.7 and 80.5 %, respectively, and the positive predictive value and negative predictive value were 77.2 and 69.0 %, respectively. When comparing to other single independent predictors, the scoring system had more satisfied predictive value (Table 3).

**Table 3:** The predictive value for the scoring system and independent predictors

Independent predictors	Sensitivity (%)	Specificity (%)	+PV (%)	−PV (%)
Size	79.8	46.3	60.4	69.1
Tumor location	36.9	80.5	66.0	55.5
Extrathyroidal extension	59.5	62.6	63.0	59.0
Margin	60.7	59.8	60.7	59.8
Display of enlarged lymph nodes	65.5	58.5	61.8	62.3
Contact of >25 % with the adjacent capsule	41.7	80.5	68.6	57.4
The scoring system	64.7	80.5	77.3	69.0

*+PV* positive predictive value, *−PV* negative predictive value

## Discussion

In our study, we listed a total of 16 potential predictors according to the clinicopathological and US features. Using the univariate analysis, we found that seven predicting factors were significantly associated with the status of central LNM. In addition, six independent predictors of tumor size (>5 mm), tumor location (the lower polo), extrathyroidal extension, margin (no well-defined), display of enlarged lymph node, and contact of >25 % with the adjacent capsule for CLNM were confirmed by multivariate logistic regression analysis.

The incidence of CLNM in PTMC patients has been reported ranging from 24.1 to 64.1 % [2, 8]. In this study, we found that the CLNM rate was 50.6 %, which was in accordance with the incidence rate.

Wada et al. [2] had reported that the location of PTMC within the thyroid was related to the prevalence of CLNM. Xu et al. [13] found that a tumor located in the lower third of the thyroid lobe was a high-risk factor of central lymph node metastasis. Zhang et al. [14] showed that the patients with a tumor located in the upper pole of the thyroid lobe conferred a lower risk for CLNM and a higher risk for lateral cervical metastasis. Similarly, we discovered that tumor location was an independent predictor for CLNM. The tumor located in the lower polo of the thyroid has a higher risk for CLNM, while the tumor located in the upper third may be a lower risk for CLNM.

Moon et al. [15] certified that PTMC with minimal ETE more frequently had CLNM than without. He et al. [16] found that extrathyroidal extension was associated with subclinical CLNM. Varshney et al. [17] declared the ETE was associated with the CLNM of PTMC. In agreement with previous results, we found that thyroid nodules with ETE were an independent predictive factor for CLNM.

Generally, no well-defined margin of the thyroid nodule has the strongest risk of malignance. Ito et al. [18] suggested that no well-defined margin was an important US feature of biologically aggressive PTMC. Also, we found that the margin was a highly independent predictor for CLNM in PTMC.

Traditionally, patients who have PTMC presenting with palpable lymphadenopathy, by preoperative US, should have cervical node dissection [2]. PTMC with ultrasonographical display of metastatic lymph nodes, especially enlarged lymph nodes, was more likely to recur in the regional lymph nodes [19]. According to the univariate and multivariate analyses, we found that display of enlarged lymph nodes was an independent predictor for CLNM.

Our study found contact of >25 % with the adjacent capsule was an independent predicting factor for CLNM. This US feature is a very important and visualized in ultrasonic examination. Many literatures suggest that the presence of contact with the adjacent capsule can provide a useful predictive information about ETE [20, 21]. According to the above analysis, about the relation between ETE and CLNM, there was an indirect relationship between contact of >25 % with the adjacent capsule and CLNM.

Many literatures confirmed that tumor size was associated with CLNM. [2, 22]. Many literatures confirmed that tumor size has significant relation with CLNM in PTMC using the cutoff value of 5 mm [13, 23, 24]. Therefore, in the present study, the tumor size of 5 mm was regarded as the cutoff value and was proved as an independent predictor for CLNM.

In this study, calcification was not an independent factor for central LNM, although it was statistically significant on the univariate analysis, which was similar to two previous reports [20, 25]. Age is known to be an important prognostic factor for patients with PTC. However, its predictive value in PTMC was uncertain [2, 23]. In our study, the gender has no association with CLNM. Similarly, Liu et al. [23] demonstrate that there was no relation observed between CLNM and gender in a meta-analysis. The association between multifocality and CLNM was still debatable [22, 23, 26]. In our study, no relevance was found between them. The main reason may be explained that the incidence rate of multifocality in the present study is very lower than other studies.

The role of pCLND was still a debate. In our opinion, those previous studies which analyzed the clinical results of pCLND had a deficiency. When assessing complication rates after thyroidectomy, most data are from high-volume endocrine surgery centers of excellence where the surgeons have more operation experience and lower complications rates [10]. In other words, the surgeons’ operation experience on CLND and hospital level had important influence on survival, postoperative region recurrence, and complications in PTMC. Hence, the issue of pCLND is likely to remain so.

If the CLNM could be diagnosed definitively preoperatively, the CLND can be easily established. However, in many patients, CLNM may not show any abnormal finding in preoperative imaging examinations due to their limitation. To improve the diagnostic accuracy, we used a combination of clinicopathologic and US characteristics to find six independent predictors for CLNM and developed a scoring system for predicting CLNM in PTMC. Using ROC curves, we identified the cutoff value of 5 points as the best score for detection of CLNM with a sensitivity of 64.7 %, a specificity of 80.5 %, positive predictive value of 77.2 %, and negative predictive value of 69.0 %. The scoring system has obvious advantages in predicting CLNM in PTMC relative to other single factor. Moreover, the predictors in this scoring system mainly come from US characteristics, which is convenient and feasible for predicting CLNM.

At present, there are many PTC-specific mutations, such as BRAF V600E and TERT promoter, which can predict the risk of CLNM in PTMC [27–29]. However, in Asia, most countries are the developing countries and their medical levels are lower than Europe and the USA. Patients often cannot afford to take more medical examination like molecular test. Therefore, in many Asian developing countries, the scoring system we raised has the advantages of simplicity and high sensitivity and specificity (compared with ultrasound) and will be helpful for surgeons to decide the essential surgical strategy for patients with PTMC, in particular the subclinical CLNM. In addition, we think that the use of this scoring system will reduce the surgical complications and develop the individual therapy for PTMC.

However, there are several potential limitations in this study. Firstly, the PTMC has very favorable prognosis and the follow-up time would be at least 10 years. Our study did not analyze data from the long-term follow-up period, such as disease recurrence and disease-free survival; thus, we cannot directly conclude that the scoring system can predict the prognosis. Secondly, this study was conducted in a cross-sectional design, rather than longitudinal observations. Thirdly, single-center verification is not accurate enough. The scoring system needs multicenter validation to check its predictive value. Further investigation with a longer duration is needed.

## Conclusions

In conclusion, we first established a scoring system for predicting CLNM preoperatively in PTMC. The scoring system of clinicopathological and US characteristics for the prediction of CLNM can be another alternative diagnostic approach when the results of preoperative diagnostic evaluation for CLNM are not adequate or obvious. The points ≤ 5 could be considered as a low risk for CLNM, and the points > 5 could be considered as a high risk for CLNM. More aggressive diagnostic approaches and prophylactic CLND are needed for the points > 5. Therefore, the scoring system will be helpful for surgeons to decide the most suitable surgical strategy for patients with PTMC, especially the subclinical CLNM.

## Abbreviations

CLND: central lymph node dissection
CLNM: central lymph node metastasis
FNAC: fine-needle aspiration cytology
pCLND: prophylactic central lymph node dissection
PTMC: papillary thyroid microcarcinoma
US: ultrasonography
